# Calcinose pseudo-tumorale chez un hémodialysé chronique

**DOI:** 10.11604/pamj.2017.28.250.14317

**Published:** 2017-11-21

**Authors:** Nadia Kabbali, Tarik Sqalli

**Affiliations:** 1 Service de Néphrologie, CHU Hassan II, Fès, Maroc; 2 Equipe de Recherche REIN, Faculté de Médecine et de Pharmacie, Université Sidi Mohamed Ben Abdellah, Fès, Maroc

**Keywords:** Calcinose pseudo-tumorale, produit phosphocalcique, hémodialyse, Pseudotumoral calcinosis, phosphocalcic product, hemodialysis

## Abstract

Pseudotumoral calcinosis (PTC) is characterized by calcium phosphate crystal deposition in periarticular soft tissues leading to the development of large calcified masses. Although PTC physiopathogeny is not completely clear, the increase in calcium-phosphorus product beyond the threshold value for precipitation as well as severe hyperparathyroidism seem to play a determining role. PTC may even cause recurrent minor joint traumas. In patients on hemodialysis, the frequency of PTC is estimated between 0.5 and 7%, according to case series. Its treatment is controversial. Surgical resection is often recommended. We here report a case of PTC in a patient on haemodialysis. The study involved a 56-year old patient on chronic haemodialysis for 9 years due to indeterminate nephropathy. The patient had suffered from gradual worsening of pain at the level of the right hip associated with difficulties in joint mobilization over the last 6 months. CT scan showed multiple lobed calcified mass, measuring 8.6 x 7.6 x 5.9cm, located very closely to the sciatic nerve, which probably explained the very painful physical symptoms. Laboratory tests showed high phosphocalcic product and hyperparathyroidism. Surgical resection was difficult due to the closeness of the tumor to vasculo-nervous elements. Our study highlights the diagnostic and therapeutic difficulties of PTC. This uncommon disease should be suspected in patients on chronic hemodialysis with periarticular calcified mass suggesting tumor.

## Image en médecine

La calcinose pseudotumorale (CPT) est une affection caractérisée par le dépôt de cristaux de phosphate de calcium dans les tissus mous périarticulaires, réalisant de volumineuses masses calcifiées. Bien que la physiopathogénie des CPT ne soit pas totalement élucidée, l'augmentation du produit phosphocalcique au-delà du seuil de précipitation ainsi que l'hyperparathyroïdie sévère, semble y jouer un rôle determinant. Le rôle favorisant de microtraumatismes articulaires répétés est également évoqué. Chez les sujets dialysés, la fréquence de la CPT est estimée entre 0,5 et 7% selon les séries. Son traitement demeure controversé. L'exérèse chirurgicale est souvent recommandée. Nous rapportons un cas de CPT survenue chez un patient hémodialysé. Il s'agit d'un patient âgé de 56 ans, en hémodialyse chronique depuis 9 ans pour une néphropathie indéterminée. Le patient présente depuis 6 mois des douleurs d'aggravation progressive au niveau de la hanche droite, avec des difficultés de mobilisation de l'articulation en regard. Le scanner alors réalisé montre une masse calcifiée, à contours polylobés, mesurant 8,6 x 7,6 x 5,9cm, présentant des rapports très étroits avec le nerf grand sciatique, ce qui explique probablement le tableau clinique très douloureux. Le bilan biologique montre un produit phosphocalcique élevé et une hyperparathyroïdie. L'exérèse chirurgicale est jugée difficile vu les rapports de la tumeur avec les éléments vasculo-nerveux. Notre observation illustre les difficultés diagnostique et thérapeutique de la CPT. L'existence de cette affection peu fréquente nous paraît devoir être rappelée, afin de l'évoquer rapidement chez un patient hémodialysé chronique devant toute masse périarticulaire calcifiée d'allure tumorale.

**Figure 1:**
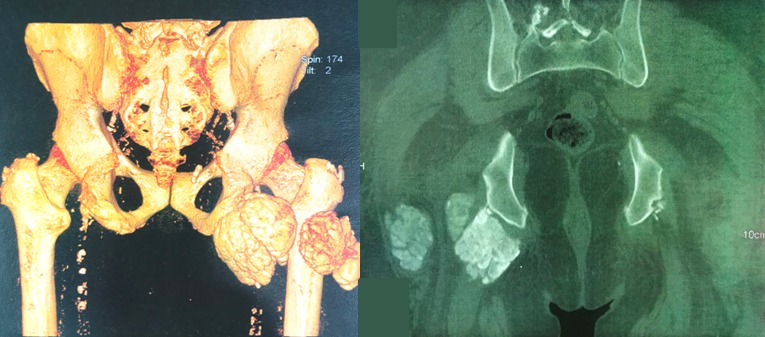
Image scannographique objectivant une masse calcifiée polylobée autour de l’articulation de la hanche droite en faveur d’une calcinose pseudo-tumorale chez un hémodialysé chronique

